# Hole diameter ratio for prediction of anatomical outcomes in stage III or IV idiopathic macular holes

**DOI:** 10.1186/s12886-020-01614-7

**Published:** 2020-08-28

**Authors:** Yue Qi, Yanping Yu, Qisheng You, Zengyi Wang, Jing Wang, Wu Liu

**Affiliations:** 1 Beijing Tongren Eye Center, Beijing Ophthalmology and Visual Science Key Lab; Beijing Tongren Hospital, Capital Medical University, 1 Dongjiaomminxiang Street, Dongcheng District, Beijing, 100730 China; 2 Casey Eye Institute, Oregon Health Science University, 515 SW Campus Drive, Portland, OR 97239 USA

**Keywords:** Idiopathic macular hole, Predictive factor, Vitrectomy, Internal limiting membrane peeling, Optical coherence tomography

## Abstract

**Background:**

To determine whether preoperative hole diameter ratio (HDR) is a predictive factor for postoperative anatomical outcome for stage III or IV idiopathic macular holes (IMHs).

**Methods:**

One-hundred and one eyes with stage III or IV IMH were included in this retrospective case series study. All cases were treated with vitrectomy combined with internal limiting membrane (ILM) peeling and room air tamponade. The macular hole (MH) minimum and maximum diameter was measured on preoperative optical coherence tomography (OCT) images. The HDR was defined as the minimum to maximum diameter ratio.

**Results:**

Eighty-one eyes (80.2%) got a Type I closure after surgery (group A). Postoperative unclosed MHs were found in 20 eyes (19.8%) (group B). The preoperative minimal diameter (703.6 ± 116.1 μm vs. 597.6 ± 120.1 μm, *P* < 0.01) and HDR (0.6 ± 0.1 vs. 0.5 ± 0.1, *P* = 0.01) were both significantly smaller in postoperative closed eyes. The closure rate of IMHs with HDR < 0.6 was significantly higher than those with HDR ≥ 0.6 (90.2% vs. 65.0%*P* = 0.002) .

**Conclusions:**

Preoperative HDR < 0.6 is predictive for a good postoperative anatomical outcome in stage III or IV IMHs.

## Background

Idiopathic macular hole (IMH) is one of the most often causes of poor vision. The estimated incidence of IMH ranged from 0.3 to 0.8% in general population [1, 2]. It may cause a small dark spot in the central vision. MH may be related with high myopia or ocular trauma, but the reason of most MHs is unknown (idiopathic) [3]. Based on the optical coherence tomography (OCT) images of macular region, IMHs can be divided into 4 stages, stage III or IV MH was considered as refractory macular hole (MH) due to the low close rate after surgery [4].

Pars plana vitrectomy (PPV) is the most popular way to treat full-thickness MH at present [5]. The main indications to perform vitrectomy in eyes with MH are as following: stage II-IV MH; decreased visual acuity (0.05–0.5) or/and visual distortion. Internal limiting membrane (ILM) peeling has been proved to increase the closure rates of IMHs [6]. It has been reported that more than 90% of IMHs closed after being treated with PPV combined with ILM peeling and inert gas or room air tamponade [7–10]. The initial closure rates of IMHs ranged from 75.6 to 100% [11–15]. However, there are still some IMHs cannot close after surgery, especially in stage III or IV IMHs. The reason remains to be elucidated.

The preoperative factors that may affect the closure of stage III or IV IMHs are great interest. These factors can help prompting surgeons to choose different operating methods preoperatively. A lot of factors have been investigated as potential predictors of final outcomes in IMH. Better surgical and functional results are associated with earlier disease stage, better preoperative visual acuity (VA), shorter duration of symptoms, younger patient age, vertical metamorphopsia and the experience of the surgeon [16–26]. With the further understanding of IMH, more factors have been found to predict the closure of IMHs. Several studies have demonstrated that the preoperative MH morphological indexes (eg. Macular Hole Index (MHI), Hole Form Factor (HFF), Tractional Hole Index (THI), Diameter Hole Index (DHI), Macular Hole Closure Index (MHCI), Central Subfield Retinal Thickness (CSRT), et al) can predict the anatomical outcomes after surgical interventions for MHs [27–33]. However, these studies are limited by their relatively small sample size. In addition, none of them aims specially at stage III or IV IMHs, which are the hardest IMHs to be healed. These parameters are also complex and difficult to calculate, limited their application values in busy real world clinical practice [16–19].

Based on our clinical experiences [11, 20, 23–26], we feel that the preoperative MH morphological characteristics are different between postoperative closed and unclosed stage III or IV IMHs. We try to find a method to quantitatively evaluate the morphological characteristics of preoperative MHs to predict the postoperative closure for stage III or IV IMHs. We hope this method is simple and easy to use in clinical work.

The objective of this study is to evaluate the ratio of minimum diameter to maximum diameter, which named as “hole diameter ratio”(HDR), as a predictive factor for the anatomical outcomes in stage III and IV MHs.

## Methods

### Patients and examinations

This retrospective, interventional case series study followed the tenets of the Declaration of Helsinki and was approved by the Institutional Review Board of the Beijing Tongren Hospital, Capital Medical University. We included consecutive patients with stage III or IV MHs who underwent PPV with ILM peeling and room air tamponade at Beijing Tongren Eye Center from November, 2016 to September, 2017 and July, 2018 to November, 2018. Before the surgical procedure, informed consent was obtained from all patients.

We excluded patients less than fifty years old, or with a stage I and II IMH, or with high myopia (axial length > 26.0 mm and/or refractive power < − 6 diopter), or with traumatic MHs, or with a history of vitrectomy or scleral buckling surgery or intravitreal injection, or with a history of uveitis, or any other fundus diseases or anterior segment diseases that may affect the outcomes.

The data of age, sex, preoperative lens status, refractive error, axial length, preoperative best-corrected visual acuity (BCVA), and OCT findings were recoded. The IMHs’ minimum and maximum diameter, HDR, were measured and calculated.

OCT (Cirrus high-definition OCT; Carl Zeiss, Dublin, CA, USA) was performed pre- and post-operatively. The anterior segments of all the patients were evaluated using slit-lamp biomicroscopy. Fundus examination was performed using binocular indirect ophthalmoscope together with fundus photography (fundus camera, TRC-50; Topcon, Tokyo, Japan). Snellen best-corrected visual acuity (BCVA), axial length of the eyeball (by IOLMaster Biometry; Carl Zeiss Meditec, Jena, Germany), and intraocular pressure (by noncontact tonometry; Full Auto Tonometer TX-F; Canon, Canada, QC) were measured for every participant.

The OCT images were reviewed and analyzed independently by two retinal specialists(Y.Q. and YP.Y.). In case of doubt, a senior retinal specialist (W. L.) was consulted and the panel discussed to reach a consensus. The multiple consecutive raster horizontal OCT scans were reviewed. A tracking system modality was used. The section with the largest tissue defects in MH was selected for measurement. The minimum diameter was measured at the nearest ends of the broken retinal tissue, while the maximum diameter was measured at the farthest ends of the retinal tissure at the same section. (See Figs. 2 and 3) Stages of MHs were determined according to both clinical features and intraoperative observations.

### Surgical technique

A standard 23-gauge 3-port pars plana vitrectomy (Constellation device, Alcon, Fort Worth, TX) was performed under local anesthesia by the same experienced surgeon (W.L.). Phacoemulsification and IOL (CT ASPHINA 509 M; Carl Zeiss Meditec Inc., Jena, Germany) implantation were first performed if necessary. Vitrectomy was performed and the posterior hyaloid was elevated and trimmed to the peripheral vitreous base in all patients. A macular epiretinal membrane was peeled if it presented simultaneously. Then the ILM was peeled off with forceps in an area of at least 2disc diameter around the MH without staining. The peripheral retina was inspected carefully and the degeneration areas or tears were photocoagulated with argon laser if they existed. The vitreous was filled with room air at the end of the surgery. Scleral incisions closed automatically. Patients were asked to stay in a prone position for at least 5 days after surgery. At postoperative follow-up examinations, measurements of visual acuity and OCT were performed at 2–4 weeks.

### Statistical analysis

Statistical analysis was performed using SPSS for Windows (version 23.0; IBM-SPSS, Chicago, IL). The results were expressed as the mean ± standard deviation (SD). Differences in the incidence rates of OCT findings between patients with or without unclosed MH were analyzed by corrected Chi-square test. The measurements of BCVA were converted into the logMAR (logarithm of the minimum angle of resolution). Mean age, duration of symptoms, axial length, BCVA, intraocular pressure and refractive error of the closed and unclosed group were compared using independent samples t-test. The discriminating power of HDR to predict closure rate after initial surgery was estimated by calculating the area under the receiver operating characteristic curve, with the area larger than 0.6 considered not determined by chance. Normal distributions of the data were checked before selecting the statistical analysis methods. The factors that may be related to the success of the initial surgery were analyzed using binary logistic regression analysis. A *P*-value < 0.05 was considered statistically significant.

## Results

### Preoperative clinical characteristics

There were 101 eyes of 101 patients with stage III (*n* = 65,64.4%) or IV (*n* = 36, 35.6%) IMH that underwent PPV combined with ILM peeling and air tamponade. No serious postoperative complications happened. 26 patients (25.7%) were men, and 75 patients (74.3%) were women. One eye (1.0%) was pseudophakic and 100 eyes (99.0%) were phakic.

### Comparison of the preoperative clinical characters of the closed and unclosed groups

Eighty-one eyes (80.2%) got a closed IMH (Type I) after one operation (defined as Group A). Postoperative unclosed IMHs were found in 20 eyes (19.8%) (defined as Group B). There were 50 (61.7%) stage III IMHs and 31(38.3%) stage IV IMHs in group A and 15 (75.0%) stage III IMHs and 5 (25.0%) stage IV IMHs in group B. Patients in group A and B did not differ significantly in age (64.1 ± 4.4 years versus 67.0 ± 4.5 years; *P* = 0.32), duration of symptoms (8.3 ± 11.1 months versus 8.3 ± 3.9 months, *P* = 0.38), preoperative BCVA (1.1 ± 0.4 logMAR versus 1.0 ± 0.4 logMAR, *P* = 0.68), preoperative IOP (15.5 ± 2.7 mmHg versus 16.0 ± 3.5 mmHg, *P* = 0.46), and axial length (23.4 ± 0.7 mm versus 22.9 ± 0.7 mm; *P* = 0.24) (Table 1).

**Table 1:** Comparison of Pre-operative Characteristics in Two Groups

	Group A	Group B	t	P
Eyes	81	20		
Age (years)	64.1 ± 4.4	67.0 ± 4.5	−0.9	0.32
Duration of symptoms (months)	8.3 ± 11.1	8.3 ± 3.9	−0.9	0.38
PreopBCVA (LogMAR)	1.1 ± 0.4	1.0 ± 0.4	−0.4	0.68
IOP (mmHg)	15.5 ± 2.8	16.0 ± 3.5	0.2	0.46
Axial Length (mm)	23.4 ± 0.7	22.9 ± 0.7	0.2	0.24
Minimum Diameter (μm)	597.6 ± 120.1	703.6 ± 116.1	−3.6	< 0.01
Maximum Diameter (μm)	1144.6 ± 182.0	1232.2 ± 186.3	−1.9	0.58
HDR	0.5 ± 0.1	0.6 ± 0.1	−1.9	0.03

Group A: eyes with closed MH; Group B: eyes with unclosed MH

BCVA: best corrected visual acuity; IOP: intra-ocular pressure

Minimum and maximum diameter of IMH was also analyzed. The mean minimum diameter was significantly smaller in group A than in group B (597.6 ± 120.1 μm vs. 703.6 ± 116.1 μm, *P* < 0.01). The mean maximum diameter was not significantly different between the two groups (1144.6 ± 182.0 μm vs. 1232.2 ± 186.3 μm, *P* = 0.58) (Table 1).

In patients with a disease duration of more than 6 months, the mean minimum diameter was significantly smaller in group A than in group B (572 ± 140 μm vs. 688 ± 89 μm, *P* < 0.01). The mean maximum diameter was not significantly different in the two groups (1032 ± 202.0 μm vs. 1195 ± 84 μm, *P* = 0.08).

### Analysis of HDR in the two groups

The mean HDR was significantly smaller in group A than in group B (0.5 ± 0.1 vs. 0.6 ± 0.1, *P* = 0.03).

**Fig. 1:**
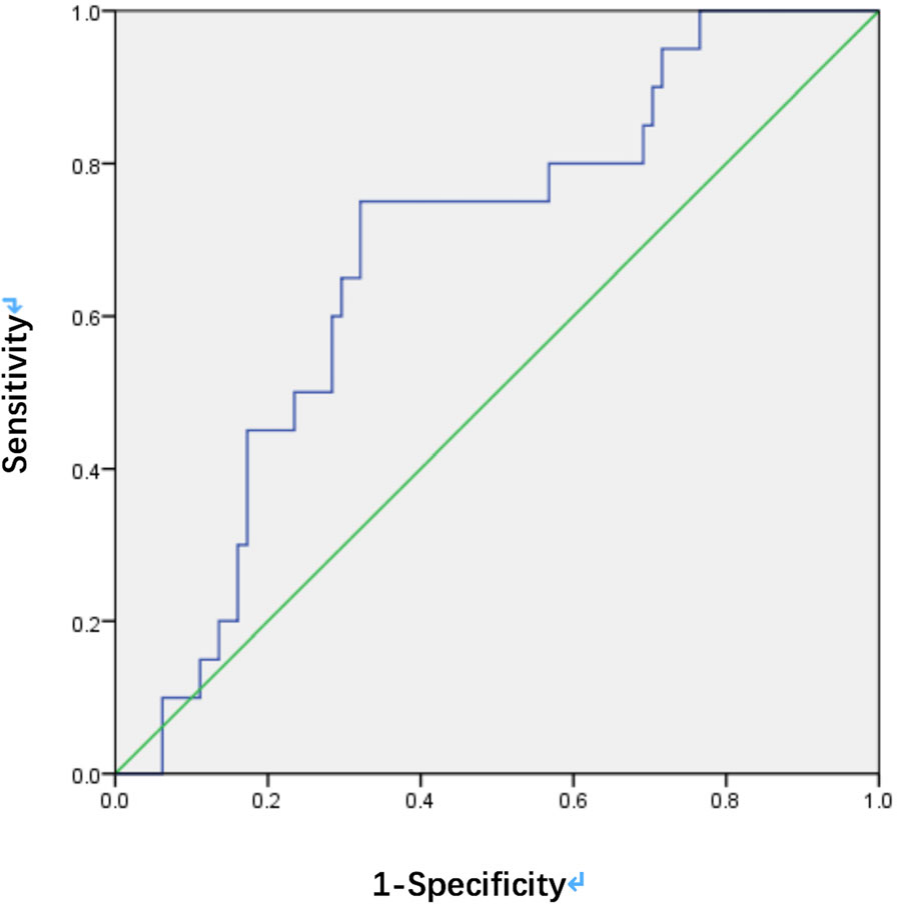
The area under the receiver operating characteristic curve of preoperative hole diameter ratio (HDR) for predicting postoperative macular hole closure. The area under the curve was 0.680 (95% confidence interval 0.559–0.801). When the cutoff value of HDR was set at 0.6, the predicting sensitivity and specificity were 75.0 and 67.9%, respectively (P = 0.01)

The area under the receiver operating characteristic curve of preoperative HDR for differentiating postoperative closed and unclosed MH was 0.680 (95% confidence interval 0.559–0.801, *P* = 0.01) (Fig. 1). When the HDR cutoff value was set at 0.6, the sensitivity was 75.0%, with the specificity 67.9%.

In patients with a disease duration of more than 6 months, the mean HDR was also 0.5 ± 0.1 in group A, significantly (*P* = 0.02) smaller than 0.6 ± 0.1 in group B. In these patients with long duration, when the cutoff value of HDR was set at 0.6, the sensitivity for predicting postoperative closure was 78.6%, and the specificity was 70.5%.

With the HDR cutoff value 0.6, we classified IMHs into A-type (HDR < 0.6, *n* = 61) and H-type (HDR ≥ 0.6, *n* = 40). There was significantly more A-type IMHs in group A than in group B (67.9% vs. 30.0%, *P* = 0.002). Overall, the mean minimum diameter of IMHs was significantly smaller in A-type than that in H-type (567.0 ± 108.2 μm vs. 697.3 ± 110.9 μm, *P* < 0.01). Subgroup analysis demonstrated In group A, the minimum diameter of A-type IMHs was also significantly smaller than that of H-type (559.0 ± 102.9 μm vs. 679.1 ± 114.6 μm, P < 0.01), but not in group B (639.7 ± 137.5 μm vs. 731.0 ± 98.7 μm, *P* = 0.11). For A-type IMHs, both the mean minimum diameter (559.0 ± 102.9 μm vs. 639.7 ± 137.5 μm, P = 0.1) and HDR (0.5 ± 0.1 vs. 0.5 ± 0.02, *P* = 0.4) were not significantly different between group A and B. Similarly, for H-type IMHs, the mean diameter (679.1 ± 114.6 μm vs. 731.0 ± 98.7 μm, *P* = 0.2) and HDR (0.6 ± 0.1 vs. 0.6 ± 0.04, *P* = 0.3) were not significantly different between group A and B. (Table 2).

**Table 2:** Comparation of two type IMHs between group A and B

		A-Type IMH	H-Type IMH	t	P
Group A	N (%)	55 (67.9)	26 (32.1)		
	Diameter^a^ (μm)	559.0 ± 102.9	679.1 ± 114.6	−4.7	0.000
	HDR	0.5 ± 0.1	0.6 ± 0.1		
Group B	N (%)	6 (30)	14 (70)		
	Diameter^a^ (μm)	639.7 ± 137.5	731.0 ± 98.7	−1.7	0.11
	HDR	0.5 ± 0.02	0.6 ± 0.04		
Mean Diameter^a^ (μm)		567.0 ± 108.2	697.3 ± 110.9	−5.9	0.000
Comparation of Diameter^a^ between group A and B	t	−1.8	−1.4		
	P	0.1	0.2		
Comparation of HDR between group A and B	t	−0.9	1.0		
	P	0.4	0.3		

*IMH* idiopathic macular hole

*HDR* Hole diameter ratio

^a^All “diameter”s in Table 2 refer to the minimum diameter of IMHs

In a multivariate logistic regression model, the post-operative closure of IMH was significantly associated with preoperative minimum IMH diameter (*P* = 0.02) and HDR (*P* = 0.04), but not significantly associated with gender(*P* = 0.97), age(*P* = 0.15), duration of disease(*P* = 0.28), preoperative visual acuity(*P* = 0.08), or axial length (*P* = 0.29).

### Comparison of closure rate of two types of IMHs

The postoperative closure rate was significantly higher in A-type IMHs than that in H-type IMHs (90.2% vs. 65.0%, *P* < 0.01).

## Discussion

The results demonstrated preoperative HDR is a predictive factor for post-operative closure of IMHs. A preoperative HDR ≥ 0.6 predicted a higher risk of unclose in stage III or IV IMHs after being treated with PPV combined with ILM peeling.

The relationships between preoperative OCT measurements and prognosis were revealed for the first time by Ip et al. at 2002 [16]. By comparing the anatomical reduction rate and recurrence rate of the IMH after operation, the authors found that IMH with a diameter under 400 μm had a higher recovery rate. Kusuhara et al. [18] proposed the concept of macular hole index (MHI, ratio of hole height to base diameter of hole) and found that MHI significantly correlated with the postoperative BCVA. However, most of the previous studies associated with MHI were limited to the IMHs under stage III. Hole form factor (HFF) [27] was defined as the ratio of the sum of the lengths of the left and right oblique sides to the base diameters of the IMH. Ullrich et al. [28] found MHs with HFF > 0.9 had a high closure rate after surgery, whereas those with HFF < 0.5 had a low closure rate. Haritoglou et al. [29] believed that higher HFF correlated with better postoperative visual outcome and the correlation coefficient between HFF and postoperative visual acuity was 0.36. Ruiz-Moreno et al. [19] proposed tractional hole index (THI, ratio of height to minimum diameter) and diameter hole index (DHI, ratio of minimum diameter to baseline diameter). They found THI correlated significantly with postoperative best corrected visual acuity, but DHI did not. Wakely L et al. [30] measured macular hole inner opening diameter, minimum linear diameter, base diameter and macular hole height and calculated the MHI and the tractional hole index (FHI). They found base diameter, macular hole inner opening and minimum linear diameter could be used to predict anatomical and/or functional success rate of macular hole surgery. Preoperative base diameter is the most useful variable in this regard. Mingwei Zhao et al. [31] proposed a method named macular hole closure index (MHCI) to predict anatomical outcome after IMH surgery. MHCI was calculated as (M + N)/BASE based on the preoperative OCT status. M and N were the curve lengths of the detached photoreceptor arms, and BASE was the length of the retinal pigment epithelial layer (RPE layer) detaching from the photoreceptors. Some studies used central subfield retinal thickness (CSRT) to predict anatomical results of MH surgery, but the results were contradictory [32, 33].

It is well known that stage III and IV IMHs are more difficult to heal [4]. However, few studies have assessed factors that affecting the healing of stage III and IV IMHs. In this study, we reviewed the records of 101 eyes treated with PPV combined with ILM peeling and room air tamponade to determine the anatomical predictive factors for stage III and IV IMHs and tried to find an easy way to evaluate it before surgery. Our results demonstrated there were no significant differences in age, duration of symptoms, preoperative BCVA, intraocular pressure (IOP) and axial length between closed and unclosed IMHs. While the minimal diameter(*P* < 0.01) and the HDR (*P* = 0.01) are significantly different in the two groups. Minimum diameter and HDR are two important predictive factors for closure of stage III and IV IMHs.

Diameter is an important factor that influences the outcomes of IMH surgery. It has been reported that IMH with a minimum diameter under 400um had a higher recovery rate [6]. Wu Liu [11] found the diameter more than 677um is a risk factors for postoperative unclosure of stage III and IV IMHs that underwent 23-gauge vitrectomy, ILM peeling, and air tamponade. In the present study, the preoperative mean minimum diameter was significantly smaller in postoperative closed IMHs than that of unclosed IMHs (597.57.09 ± 120.14 μm vs. 703.60 ± 116.13 μm, *P* < 0.01). The results are consistent with previous studies [6, 11].

HDR (hole diameter ratio) is defined as the ratio of minimum diameter to maximum diameter in this study. It reflects the magnitude of pulling force in the tangential direction of the IMH. The mean HDR was significantly lower in closed IMHs than that of unclosed ones (0.5 ± 0.1 vs. 0.6 ± 0.1, *P* = 0.03). Ratio of minimum diameter to maximum diameter has been mentioned as diameter hole index (DHI) in previous studies [19]. While in those studies, no relationship was found between the ratio and postoperative anatomical/functional outcome. Several reasons may explain the discrepancies. First, the study patients were different. All stages of IMHs were included in previous studies, while only stage III and IV IMHs were included in current study. Stage III and IV IMHs are refractory IMHs. The diameters are larger and the pulling force in the tangential direction may play a more important role in these IMHs. In stage III/IV IMHs, posterior vitreous detachment happened, which means the tangential force become the main force affecting IMH. Therefore, HDR may be more suitable for evaluating stage III/IV macular holes. Second, sample size is different. In previous studies, the sample size was mostly below 50, which was relatively small. The sample size of this study is more than 100. We feel more confident with the relatively large sample size in the current study. In addition, the tamponades, the surgeon experiences and techniques may all influence the surgical results and have an impact on study conclusions.

**Fig. 2:**
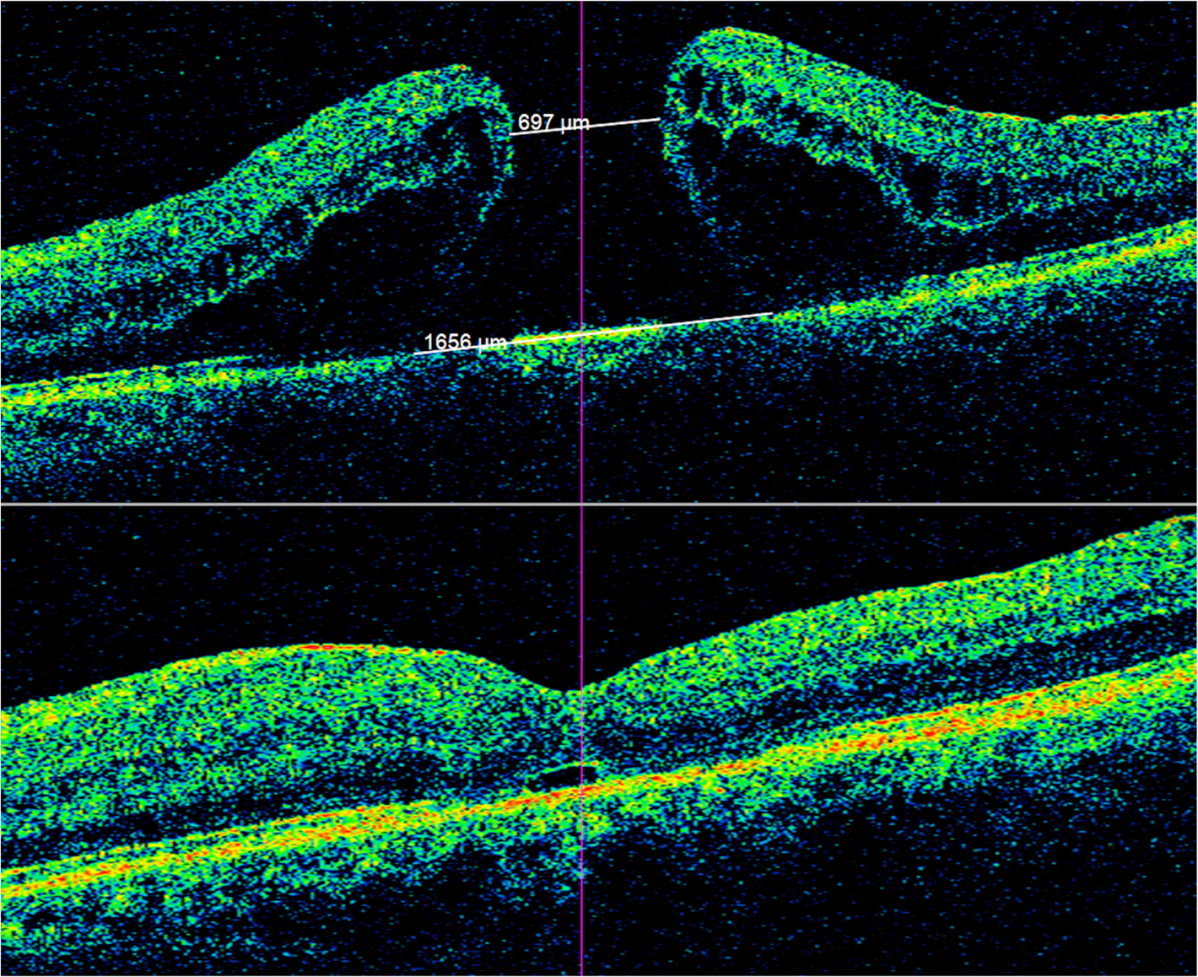
This is a Chinese patient (60–70 years old) with an A-type idiopathic macular hole. The minimal diameter was 697 μm and maximum diameter was 1656 μm. The diameter of his macular hole was “large”, but the hole diameter ratio was less than 0.6. The MH was closed at postoperative 1 month

**Fig. 3:**
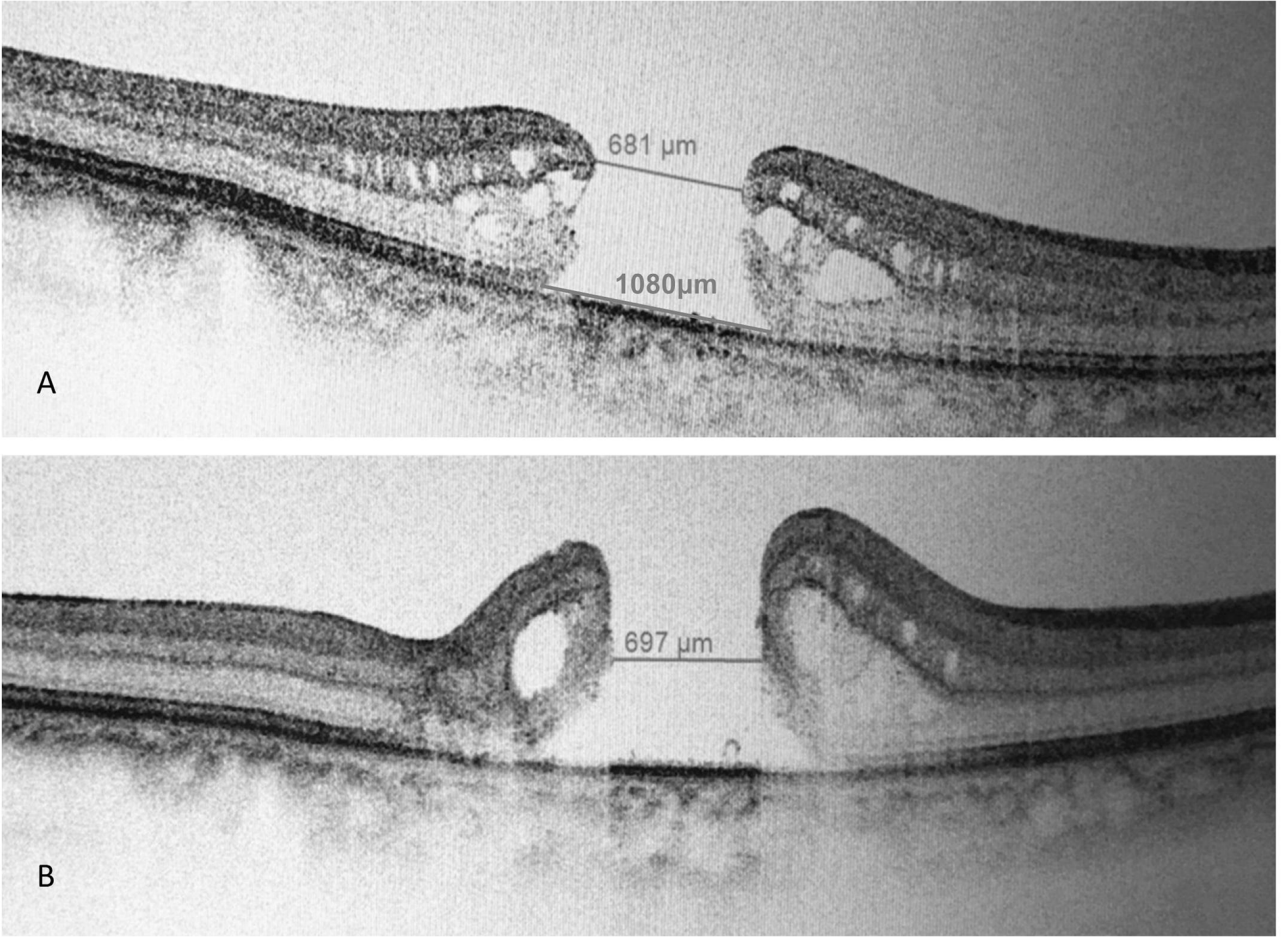
This is a Chinese patient (60–70 years old) with a H-type idiopathic macular hole. The minimal diameter was 681 μm and maximum diameter was 1080 μm. The preoperative diameter of her macular hole was similar with the patient in Fig. 2, but the hole diameter ratio was more than 0.6. The macular hole was unclosed at postoperative 3 months

The closure rate of stage III/IV IMHs is significantly higher in A-type IMHs (with HDR < 0.6) than in H-type IMHs (with HDR ≥ 0.6) (90.2% vs. 65.0%, *P* = 0.002), suggesting the anatomical results can be well predicted by preoperative HDR. The letter “A” and “H” simulate the shape of the two type IMHs. This classification may enable us to judge the possible postoperative prognosis simply and quickly according to the preoperative IMH morphology characteristics. An A-type stage III and IV IMH with a smaller diameter is relatively easy to close. While for large IMHs with a “H” shape (HDR ≥ 0.6), it’s harder to close (Figs. 2 and 3).

Disease duration is also a well-known factor affecting MH closure. In this study, the included stage III/IV IMHs had a longer course of disease than stage I/II IMHs. In addition, due to the extremely high volume of patients and operations, the time of waiting for surgery was long in our eye center (patients usually need to wait for over 6 months to be scheduled for surgery after they are diagnosed in clinics). The mean disease duration of the current study is about 8.3 months in both of 2 groups. This is longer than previous studies [16–25]. It has been reported that the IMHs with disease course more than 6 months is more difficult to close than that of less than 6 months [11]. Therefore, we did a subgroup analysis for those patients with a disease course longer than 6 months. The results are consistent with the overall results. We also analyzed the relationship between the closure of MH and gender, age, course of disease, preoperative visual acuity, ocular axial length using a multivariate model. The results showed that these factors had no significantly relationship with the closure of IMHs in current study. These results suggested that HDR may be an independent predictor for closure of stage III/IV IMHs.

We acknowledge there are some limitations in the current study. Firstly, it is a retrospective case series study, rather than a prospective cohort study. Second, we only analyzed the relationship between HDR and anatomical outcomes. The correlation between HDR and functional outcomes need to be studied further in future. The strength of the present study included a relatively large sample size of stage III / IV IMHs with a long disease duration. These IMHs are usually more difficult to close compared to stage I/II IMHs or those with a shorter duration. Therefore, finding predictive factors for these IMHs are clinically more important.

## Conclusions

In conclusion, HDR < 0.6(A-type IMH) can be a predictive factor for a good anatomical outcome in stage III or IV IMHs after vitrectomy combined with ILM peeling and air tamponade. It can be easily calculated and intuitively noticed on preoperative OCT images and may help doctors and patients to predict the surgical prognosis for stage III or IV IMHs.

## Data Availability

The datasets used and/or analyzed during the current study are available from the corresponding author on reasonable request.

## Abbreviations

HDR: Hole Diameter Ratio
IMH: Idiopathic macular hole
ILM: Internal limiting membrane
MH: Macular hole
OCT: Optical coherence tomography
PPV: Pars plana vitrectomy
VA: Visual acuity
MHI: Macular Hole Index
HFF: Hole Form Factor
THI: Tractional Hole Index
DHI: Diameter Hole Index
MHCI: Macular Hole Closure Index
CSRT: Central Subfield Retinal Thickness
BCVA: Best-corrected visual acuity
IOL: Intra ocular lens
SD: Standard deviation
logMAR: logarithm of the minimum angle of resolution
FHI: Tractional Hole Index
